# Challenges in the management of people with heart failure with preserved ejection fraction (HFpEF) in primary care: A qualitative study of general practitioner perspectives

**DOI:** 10.1177/1742395320983871

**Published:** 2021-01-05

**Authors:** Muhammad Z Hossain, Carolyn A Chew-Graham, Emma Sowden, Tom Blakeman, Ian Wellwood, Stephanie Tierney, Christi Deaton

**Affiliations:** 1 Faculty of Medicine and Health Sciences, School of Medicine, Keele University, Keele, UK; 2 NIHR School for Primary Care Research, Centre for Primary Care and Health Services Research, University of Manchester, Manchester, UK; 3 Primary Care Unit, Department of Public Health and Primary Care, University of Cambridge, School of Clinical Medicine, Cambridge, UK; 4 Nuffield Department of Primary Care Health Sciences, University of Oxford, Oxford, UK

**Keywords:** General practitioners, primary care, heart failure, HFpEF, qualitative methods

## Abstract

**Objectives:**

To explore the perspectives of general practitioners (GPs) on the identification and management of people, including those from ethnic minority groups, with Heart Failure with Preserved Ejection Fraction (HFpEF).

**Methods:**

Qualitative study. Semi-structured, face-to-face or telephone interviews and focus groups were conducted with 35 GPs in England, which were audio-recorded and transcribed verbatim. Framework analysis was used to manage and interpret data.

**Results:**

Themes presented reflect four inter-related challenges: GPs’ 1) lack of understanding HFpEF, impacting on 2) difficulties in communicating the diagnosis, leading to 3) uncertainty in managing people with HFpEF, further hindered by (4) discontinuity across the primary/secondary interface. All were considered more challenging by GPs when managing people from different cultures and languages.

**Discussion:**

HFpEF is not well understood by GPs, leading to diagnostic difficulty, management uncertainty and potential inequity in care offered. People with HFpEF are seen as complex, with multiple long-term conditions and requiring personalised care. Challenges in their management occur across the healthcare system. This study has identified learning needs for GPs around identification and on-going support for people with HFpEF in primary care. It will contribute to the development of more flexible and patient-centred pathways across the primary/secondary care interface.

## Background

Heart failure (HF) has a major impact on affected individuals, their families and healthcare services. There are an estimated 9,20,000 people living with HF in the United Kingdom (UK) and HF is the cause of over 5% of all emergency hospital admissions in the UK.^1^ HF can result from cardiovascular disease leading to heart muscle dysfunction. Many older people with HF also live with multiple comorbid conditions which require specialised health and social care services, with support to meet complex individual needs and to maintain quality of life. In the UK, the current financial cost of HF is estimated at £2 billion a year, borne by the National Health Service (NHS), local authorities and families.^2^

Historically, Heart Failure with Preserved Ejection Fraction (HFpEF) was referred to as ‘diastolic HF’ or ‘HF with normal ejection fraction’. HFpEF is normally defined as HF with an ejection fraction ≥50% and key findings of diastolic dysfunction and/or structural heart disease on echocardiogram. However, there is no universal agreement about the precise ejection fraction or parameters used to determine diastolic dysfunction and other findings in practice and clinical trials, although more robust diagnostic pathways are proposed.^3^ There is now a preference for the term HFpEF.^4^ As a result, non-specialist healthcare providers may not be confident in the diagnosis and management of HFpEF.

Approximately half of patients with HF will have a preserved ejection fraction (HFpEF).^5^ This is less well understood and less easily diagnosed than HFrEF (reduced ejection fraction).^6,7^ The diagnosis of HFpEF is challenging particularly at an early stage of the illness where symptoms are nonspecific and can be caused by numerous other non-cardiac conditions, such as chronic lung disease, anaemia, and chronic kidney disease (CKD).^8^ Furthermore, HFpEF in older adults can be comorbid with obesity, diabetes and hypertension^9,10^ and the pathophysiology and aetiology of HFpEF remains poorly understood. The exact pathophysiological mechanisms behind HFpEF are not fully understood, but likely to be multifactorial. Notwithstanding, Black, Asian and Minority Ethnic (BAME) groups have a higher prevalence of obesity, hypertension, heart disease and diabetes than the general population.^11–14^ Limited evidence shows that among all BAME groups, South Asians are thought to be at greater risk of developing HFpEF than African-Caribbean groups.^15^

The prognosis and survival of people with HFpEF is poor; nearly 40% of HFpEF patients die within 5 years following discharge from hospital for heart failure.^8^ HFpEF has been labelled a ‘stealth syndrome’,^16^ and a better understanding of its pathophysiology and management is considered an urgent priority for primary care professionals.^17^ Epidemiological trends in the United States (US) have shown hospitalisations for HFpEF are increasing.^18,19^

Within the last decade, NHS England published two significant strategies - the Five-Year Forward View^20^ and the NHS Long Term Plan,^21^ which outline required changes within the NHS to deliver best care. Relevant to the management of people with HF is the ambition to create pragmatic and practical systems by integrating primary and secondary care teams, with more care provided locally to manage people with multimorbidities, and the need to offer personalised care.^22^ The NHS Long Term Plan sets out the priorities and ambitions for personalised care, which is one of the five major, practical changes to the NHS that will take place over the next five years. Also, the NHS has announced its large-scale plan to support the rollout of personalised care 2.5 million people by 2023/24 and then aim to double that again within a decade. People with HF need to play an important part in managing their health. However, managing HF is a challenging task which requires knowledge, skills and confidence, to better manage their health and wellbeing. Personalised care is where people have more choice and control over how their health and care needs are met. It recognises that people themselves can sometimes be the best integrators of health and care. With personalised care, people are more involved in the decision-making process, and should be supported to talk about the things or outcomes that matter most to them, and what is the best course of action to achieve these outcomes. The result is better health and wellbeing for them, plus more effective and collaborative services between interface used in HF management in which patients and clinicians identify and discuss problems caused by or related to the patient's condition, and develop safe and effective care. Rapid referral from primary care for investigation and responsive specialist care were key ambitions of the NHS Five-Year Forward View^20^ with flexibility and high-quality communication between primary and secondary care services.

The 2018 National Institute for Health and Care Excellence (NICE) guideline suggests that primary care teams should take responsibility for the routine management of patients with HF once they have been diagnosed, with management optimised by the specialist HF team.^23^ However, unlike for HFrEF, there is inconsistency in the availability of specialist services for patients with HFpEF in the UK.^24^ Given the uncertainty surrounding this patient group, HFpEF remains under-diagnosed in primary care;^25,26^ and management lacks an evidence-base for specific pharmacological therapy. In addition, Quality and Outcomes Framework (QOF) indicators included in the contractual requirements for general practices in England are not required to identify which type of HF people have to establish a register.^27,28^ It is important to explore the perspectives of GPs on identification and management of HFpEF, and the support they need, as part of a drive towards optimising care for this patient group.

## Aim

This study was conducted within a larger programme of work, the Optimise-HFpEF study,^29^ which aims to improve the management of HFpEF.

The purpose of this qualitative study was to explore GPs’ experiences of identifying and managing people with HFpEF in primary care. In doing so, we sought to understand any additional challenges encountered by GPs when managing HFpEF in people of BAME backgrounds.

## Methods

Qualitative methods, incorporating semi-structured interviews and focus groups were used. Ethical approval was obtained from Health Research Authority (HRA) and Research Ethics Committee 17/NE/0199. The study was supported by a Patient Advisory Group (PAG).

## Recruitment

GP participants were recruited from general practices within three regions in England (the North West, East of England and the West Midlands), to ensure sampling of practices working with different systems and providing care in a range of areas, to different populations. This enabled us to determine commonalities and differences among diverse providers and regional healthcare systems. Participants were recruited with support from the National Institute for Health Research Clinical Research Networks (NIHR CRN). Purposive sampling was used to identify and select the GP participants by criteria, such as gender, a range of years of experience in general practice, partnership status (GP partners, salaried, and locums). Letters of invitation, participant information sheets, informed consent and expression of interest forms were sent to practices by the NIHR CRN. Interested GPs returned the expression of interest form in pre-paid envelopes or could phone/email the study researchers directly. Potential participants were then contacted by the researchers (MH, ES and IW) to explain the study and respond to questions. Informed consent was obtained prior to face-to-face interviews and focus groups. Where interviews were conducted by telephone, GPs were asked to sign the consent form, then return by post or e-mail to the researcher.

## Data collection

Data were collected through semi-structured interviews (n = 35) and focus groups (n = 1). Interviews were conducted either face-to-face at a place of the participant’s choosing (usually their practice) or by telephone, by MH, ES and IW (experienced health service researchers) between October 2017 and July 2019. One focus group with eight GPs was conducted (facilitated by CD & IW), as a pragmatic approach at one general practice, for convenience and to enable an interactive discussion among the GPs.

A topic guide was developed, based on a review of relevant literature, the team’s knowledge of HFpEF, and with input from a PAG (see online Appendix 1). Interviews and focus group followed the same topic guide which provided a frame of reference, rather than an inflexible structured process;^30^ researchers could probe areas raised by participants. In fact, it is worth mentioning that during a few interviews the sequencing and wording of the topic guide and questions were also modified by the interviewer based on the actual research interview experiences to best fit the study participant and interview context. Interviews and focus group were digitally recorded with permission and transcribed verbatim. Data collection ceased once data redundancy occurred, whereby additional participants did not provide key new information.^31,32^

## Data analysis

Framework analysis^33^ was used to analyse the data – a matrix-based format that facilitated sharing data as a team. Although focus groups and individual interviews had been conducted by a team of research associates (MH, ES, IW), all team members were involved in analysis as a team by regular team meetings (face-to-face and by telephone), with discussion of the coding and codebook. All research associates kept a research diary to record their reflective notes during the interviews and thoughts about analysis throughout the process. Familiarization involved researcher carefully read and reread the interview transcripts, their reflective notes and re-listening all the interviews. Initially, two researchers from each region independently coded the first few transcripts and circulated the codebook to the researchers of all three regions. The PAG was also involved in reviewing the analytical themes and offered alternative viewpoints to ensure themes were reliable and self-evident. Regionally, after coding a few transcripts, all regional investigator team members involved in coding met for a two-day long meeting. The team members compared the labels they applied and agreed on a set of codes to apply to all subsequent transcripts which were grouped together into categories and developed as a working analytical framework. Subsequently, three research associates (MH, ES, IW) coded a few more transcripts and virtually met again to discuss and revise the initial framework to incorporate any refined and new codes. The method of refining and applying the analytical framework was a recursive, rather than linear, process where researchers moved back and forth as needed until no new codes were generated. Researchers then applied the final analytical framework to each transcript using the QSR NVivo 12 software.^34^ For example, all the transcripts were divided between the three researchers and later imported into NVivo for indexing. Following that researchers methodically re-read each transcript, highlighted each significant excerpt, selected, and assigned a suitable code, systematically searched for patterns from the final analytical framework to generate full descriptions. After all the data had been coded using the analytical framework, the researchers summarized the data in a matrix for each theme using Microsoft Excel sheet. The matrix comprised of one case row per participant and one column per code. Four major themes but no sub-themes were then generated from the data set. Themes were generated from the data set by reviewing the matrix and making connections within and between participant and categories. This process was influenced both by the original research objectives and by new concepts generated inductively from the data. Therefore, researchers perceived that creating any sub-themes from the data would not offer any additional justification about what was taking place inside the data. However, assuming any additional sub-themes could be logged mechanically, the automated task of creating sub-themes would reduce the number of major themes. As a result, this process would make the utilization of ‘sub-theme’ in place of major themes rather redundant. To ensure rigour and credibility of the research findings, we adhered to the COREQ checklist^35^ (see online Appendix 2). 

## Results

A sample of 35 GPs from across the three study regions were recruited from 19 practices. GP demographics are shown in Table 1.

**Table 1.** Demographics of GPs interviewed.

Demographics of GP Participants	
Number of GPs interviewed	35
Gender	Male: 19
	Female: 16
Type of GP	Partner: 26
	Salaried: 8
	Locums: 1
Years working in general practice	Median: 10 years (Range: 1 to 18 years)
Practice location	Urban: 20
	Sub-urban: 9
	Rural: 6

Interviews lasted between 17 and 51 minutes (mean length 31 minutes); 10 were conducted face-to-face, 17 by telephone. The focus group was face-to-face and lasted approximately one hour.

**Figure 1.**
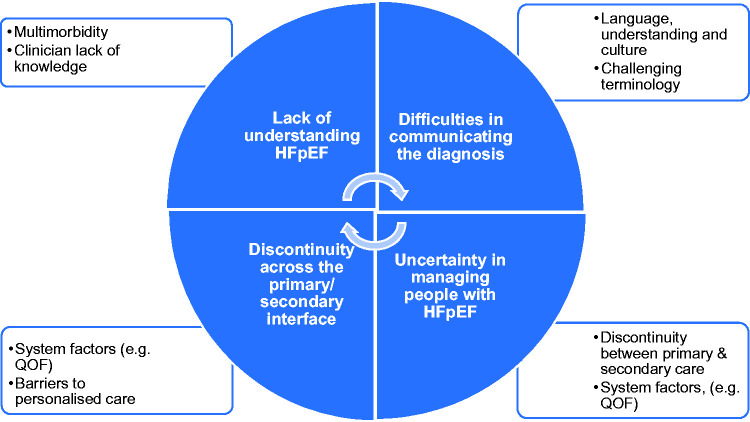
HFpEF GP model.

Themes presented reflect four inter-related challenges: 1) GPs’ lack of understanding HFpEF, which complicated 2) difficulties in communicating the diagnosis, and led to 3) uncertainty regarding patient management, further hindered by 4) discontinuity across the primary/secondary care interface. These themes were considered more challenging by GPs when managing people with cultural and language differences. The inter-relationship of themes was developed into a model, which is illustrated in Figure 1 (see discussion).

Each GP participant was allocated a unique study code number to protect their anonymity. The themes identified are illustrated with extracts identified by GP and assigned study number below.

### Lack of understanding HFpEF

The majority of GP participants described their lack of knowledge and understanding of HFpEF. Some GPs suggested that HFpEF was identical to the generic term ‘heart failure’, and most felt unable to describe its pathophysiology:

*I guess no, basically, I don’t understand the difference or how they would differ from normal heart failure patients or how I should manage them differently.* GP-17, female, years of practice 7

GPs described trying to make sense of a set of symptoms which may be attributable to HFpEF:

*I think, clinically, they all present quite similar [mmm]; leg swelling; breathlessness and other things that are quite similar. […] From what I understand, this is quite a new form of categorisation of heart failure. It’s not something I’m very familiar with. […] I will leave something like the differentiation of this to secondary care doctors.* GP-29, male, years of practice 5

Participants reflected that terms seem to be changing constantly. The lack of clear consensus on its definition and features, which would help with diagnosing HFpEF, made it harder for GPs to understand the condition clearly:

*I think it would be helpful to clarify the language … because a few years ago … so they used to talk about cor pulmonale in the old days, then they started talking about diastolic dysfunction, and now they’re talking about heart failure with preserved ejection fraction.* GP-20, female, years of practice 18

Only three GPs disclosed that they had looked up the meaning of HFpEF prior to the interview. Some also posed questions throughout their interview about the nature of HFpEF:

*… the first time I came across the term, to be honest with you – because I, I understand it’s slightly synonymous with diastolic dysfunction, isn’t it?* GP-33, male, years of practice 10

The majority of GPs described further challenges to making sense of HFpEF in BAME groups because of their increased risk of developing diabetes and other comorbid condition which were seen to increase the complexity of management:

*… multiple comorbidities, you know, they might, might have more diabetes; earlier cardiac disease; erm, chronic kidney disease, so you’ve got to manage those.* GP-27, female, a locum GP, years of practice 5

### Difficulties in communicating the diagnosis

Their own uncertainties and a lack of understanding about the diagnosis of HFpEF meant that most GP participants reported difficulties in communicating the diagnosis to patients. In addition, information about HFpEF was thought to impact negatively on patients. The majority GPs tried to balance sharing some information on HFpEF with the need to avoid inducing fear:

*Because it’s a new thing and we didn’t as doctors know much about it. So, I think as a doctor you have to try and not to give people too much information that’s going to scare them but at the same time you never withhold anything that they should know about.* GP-18, male, years of practice 14

GPs varied in whether they would use the terms ‘heart failure’ or ‘HFpEF’ or use euphemisms in discussions with patients, with some employing lay terms such as a ‘pump’ to explain the diagnosis. Hence, whilst some GPs preferred to inform patients of a specific HFpEF label, others were less willing to do so for fear of upsetting patients by telling them that their heart was not pumping well or ‘failing’. This then contributed to difficulties in explaining what management could be offered:

*I think the diagnosis of heart failure; whichever type of heart failure it is, it’s quite a hard conversation to have with patients because it's quite a dramatic term, your heart is failing […] talking about that with patients can be difficult and I think there’s even more complexity to that with HFpEF patients because you say, “well, you have what we call heart failure, but actually it's pumping alright, your heart, but what we know is these other things aren’t working as well, but we don’t actually have that much evidence about what will help, what will improve things.”* GP-16, female, years of practice 11

The majority of GPs described how such difficulties were magnified in certain groups such as patients from a BAME background, with barriers due to language differences adding an extra challenge to communicating the diagnosis of HFpEF:

*There was a high ethnic minority population and quite a lot of non-English speaking […] you try and tell people about these things and you’ve got the language barrier. So you’ve got so many challenges there to actually, not terrify the patient but make them understand what’s going on…* GP-33, male, years of practice 10

Difficulties developing a dialogue about HFpEF were compounded by perceived fear and stigma associated with HF in some BAME groups:

*I think with older ethnic minority patients my experience is that they fear being told that they have heart problems […] It’s not necessarily something they want to discuss with you. I think maybe it’s perception that maybe in years previously it’s not something that you would ever recover from…nobody minds talking about their diabetes because they all have it, whereas there’s a stigma attached to cancer and a stigma attached to heart problems.* GP-19, female, years of practice 15

However, only four GPs said that, regardless of ethnic background, conveying the diagnosis of HFpEF to patients was still complex due to the associated poor prognosis:

*I guess even though you say – you mention heart failure, people don’t still understand the seriousness of heart failure … So when explaining that, that is a challenge, isn’t it? How to say … you know, er, it is a gradual deteriorating disease.* GP-7, female, years of practice 3

### Uncertainty in managing people with HFpEF

The majority of GPs emphasised that people with HFpEF are a heterogenous group that is more likely to live with comorbidities. Treating the underlying comorbidities becomes the priority, rather than treating ‘HFpEF’ itself:

* … you can’t really have a one size fits all thing. So the aim of treating it, for me, is symptom relief but it’s also about managing the underlying condition. So if you’re diabetic we’ll take optimal diabetes management and similarly if they’ve got lung disease or COPD – and you often see HFpEF with that.* GP-22, male, years of practice 17

Half of GPs differed as to how much they felt they should be involved in the management of people with HFpEF, and where their role started and ended. Most highlighted the need for timely specialist support when the patient’s symptoms were not under control:

*I would manage them. But it depends how symptomatic they are. If they are ill and unstable they will go to secondary care. It depends on the comorbidities, their age and everything.* GP-20, female, years of practice 18

More than half of GPs described frustration when trying to access care for people with HFpEF. They stressed that they wanted to manage those with the condition with an integrated approach, following robust care pathways to meet patients’ needs from diagnosis through to end-of-life care. GPs suggested that optimising HFpEF management requires coordination with members of a multi-disciplinary clinical team. However, the specialist single condition focus may conflict with the more holistic approach in primary care, and consideration of age and other conditions was essential:

* … there has been more of a trend towards being uni-system within the hospital, …you know, the ideal cardiological solution for them may not be the holistic solution for them. And sometimes, getting someone senior enough to see that and make that decision can sometimes be a bit tricky.* GP-9, male, years of practice 12

More than a third of GP participants also described how the QOF, with its focus on targets for prescribing for HFrEF, would guide the management of all patients with HF, adding that meeting QOF indicators could preclude establishing other systems of management:

*…we tend to, I’m afraid, be led by QOF, so you know, they will be reviewed and are they on their beta blockers and ACE inhibitors and, but not in, we don’t have a, sort of, heart failure clinic, as such, no.* GP-1, female, years of practice 6

Additional complexity in management was encountered in BAME groups as some GPs admitted that they knew little about the beliefs, lifestyle, diet of people from different ethnicities. This made it difficult to give advice about lifestyle and make suggestions for change:

*And the other cultural differences are things like erm, directing things, so sometimes erm, see patients who have quite unhealthy diets and maybe sort of Asian backgrounds and things, erm, and it can be difficult to erm, sometimes difficult to advise about to make their diet healthier, partly because I think an ignorance on my part.* GP-30, female, years of practice 16

Nearly half of GPs also felt that the organisation of health services caused additional barriers to people from BAME groups:

*…if they don’t speak English, then first of all the diagnosis; the management; explaining the treatment plan to them; ensure they know how to follow up and also simple things like getting them seen by secondary care. If they send an appointment in the post, they don’t turn up because they may not know what to do. It’s simple things like that.* GP-11, female, years of practice 8

### Discontinuity across the primary/secondary interface

The majority of GPs emphasised that effective communication between primary and specialist teams was vital for the planning and delivery of appropriate care and management throughout the HFpEF journey. However, some felt that the fragmentation of care, and lack of clear care pathway could leave patients, and themselves, unsupported:

*Some might follow them up but some might just say, ‘well, asymptomatic, this is the diagnosis, managed by GP’ or some might say, ‘well erm … might have a follow up and then refer back.’ So it does – I think it does vary upon the clinician involved in secondary care as to their treatment plans.* GP-6, male, years of practice 7

Perceived lack of communication from the specialist team back to GP was a source of frustration. GPs voiced concerns related to the content and format of the communication; speed was another area of contention:

*I can write a letter but it will take a long time to get a response to that letter and sometimes I’d never get a response to that letter or the response might be, okay well I’ll see them in clinic and then their appointment time is going to be three to four months minimum.* GP-14, female, years of practice 11

GPs expected to receive the diagnosis and management information about HFpEF in a clear and concise manner, including the use of less medically ambiguous jargon. Some GPs stated that the specialists’ letters included little information about the outcomes of their clinical examinations and investigations:

*Are they detailed letters? Sometimes, not always, sometimes they just say, oh, your patient has been seen, echocardiogram just showed mild left ventricular dysfunction, so I don’t know what mild means, you know, whether, have they got anything else like, what’s the ejection fraction but if they’ve got preserved, or if they’ve got preserved left ventricular erm, ejection fraction … Not always clear, mainly we can document it they’ve got heart failure.* GP-20, female, years of practice 18

Only a few GPs suggested that information following a hospital admission or outpatient appointment was not always received in a timely manner, and that appropriate information about the need for proactive follow-up was not provided. Some GPs suggested that cardiologists had an unrealistic expectation of what can be done in primary care:

*Sometimes, it can be a little bit, you know, “Oh, just do this and just do that,” and you think, “Oh, for goodness sake, how much time have we got?” You know, or, and titrate upwards and stuff, you know, it takes a lot to titrate things up. We try, but, you know, you know, balancing these things, because it can be, certainly if you’re changing diuretics and things, you know, you have to do them quite regularly, really. You can’t just leave them for five weeks…* GP-30, female, years of practice 16

Another subgroup of GPs also described uncertainty over where the responsibility for managing people with HF lay, and inadequate arrangement for follow-up in specialist care:

*And there are some broader irritations in terms of the general practice, secondary care relationship in terms of who's taking responsibility for some of the follow-up stuff […] it's not always as clear as it should be, despite the new set of contracts that have come out of the, you know, the hospital should follow up hospital tests, they don't always quite understand that.* GP-21, male, years of practice 9

A minority of GPs reported swift communication from cardiology teams and HF clinics, with a management plan outlined. GPs highlighted the importance of building good relationships across the interface to facilitate effective communication. These established relationships can then have a positive impact on patients’ overall health and well-being as part of their HF management in primary care:

*…we have erm quite close ties with our local cardiologists…when the patient is seen in the cardiology clinic, cardiologists will normally write to us fairly quickly erm and even sort of you know, fax over erm, something on the same day if there’s anything urgent, any medication changes that need to be made erm, from our end.* GP-15, male, years of practice 13

## Discussion

### Summary of findings

This study has highlighted GPs’ perspectives on identifying and managing people with HFpEF including communication about the condition, organisation of care, and the primary/specialist care interface. GPs reported that making and communicating the diagnosis of HFpEF to patients was challenging due to limitations in their own understanding, the complexity of patients with multimorbidity, and a lack of clarity in language about HFpEF. GPs valued input from specialist care, but organisational barriers were reported, including poor communication between primary and specialist care. All challenges were perceived to be greater when managing people with cultural and language differences, thought to be more common among BAME communities. GPs described uncertainty of the boundaries of their role in HFpEF management, and the support available from specialist care. Thus, it is likely that patients have different experiences and standards of care. GPs acknowledged that a holistic approach to people with HFpEF is required, but this needed an infrastructure to provide personalised care for this patient group. We summarise our findings as a model in Figure 1 above.

### Comparison with previous literature

Our findings highlighted that GPs had limited knowledge about HFpEF and were hampered in their role by a lack of unified approach to defining HFpEF; as described by Upadhya & Kitzman.^36^ Diagnostic uncertainty around HFpEF is common.^37–39^ GPs reported that diagnosis of and prognosis for HFpEF was challenging; in part, reflecting the fact that the condition lacks a simple explanation.^4,40^ This resulted in frustration from GPs who felt unable to respond to a patient’s specific questions about the diagnosis, prognosis and treatment of HFpEF.

Patients present first to their GP with symptoms and signs which may represent HF. Previous research has documented that the path to diagnosis varies and that opportunities for earlier diagnosis may be missed.^25^ Challenges in diagnosis are further exacerbated by the low awareness of HFpEF within primary care, and greater diagnostic difficulty. Timely and accurate diagnosis of HFpEF along with control of comorbidities are vital since treatments can alter prognosis as well as optimise symptoms. Non-drug interventions such as managing fluid status, cessation of smoking, lifestyle interventions of limiting alcohol intake, eating a high-fibre diet, controlling blood pressure, and exercise training can improve HFpEF and quality of life. Therefore, a precise and timely diagnosis of HFpEF can identify patients needing management and ensure that they received appropriate treatment. Most people with HFpEF are managed in primary care in the UK,^37^ with guideline recommendations for management of comorbid conditions and fluid overload.^41^ The 2018 NICE Guideline^23^ on Chronic Heart Failure recommends that patients with HF are managed in primary care once they are stabilised by the specialist multidisciplinary team. However, surveys of specialist HF practices note that 60–80% report seeing patients with HFpEF,^24,42^ and only 53% of community services follow patients with HFpEF.^42^ Thus, as findings from our interviews reflect, GPs may receive less support from specialist services in the diagnosis and management of HFpEF than they would like.

Sharing information with patients about the diagnosis of HFpEF was challenging for GPs as reported in previous studies.^37,43–47^ Some GPs in our study used euphemisms rather than the term ‘heart failure’. This explanation of the heart as a ‘pump’ was reported in a previous study,^48^ which found that patients were less anxious when such euphemisms were used.

GPs suggested that the diagnosis and management of people with HFpEF requires specialist input. However, the primary/secondary care interface was not always easy to navigate. Previous studies confirm that effective and efficient communication between primary and specialist teams impacts on service delivery and affects patient satisfaction.^49,50^ In line with existing evidence,^51^ GPs in the current study expressed increasing frustration when communication difficulties experienced by GPs were not acknowledged by the HF specialist team. Aims of the NHS Five Year Forward View^20^ and the NHS Long Term Plan^21^ include offering personalised care, supported by enhanced interprofessional collaboration and maximum utilisation of digital technologies. Advanced and safer use of digital communication and information between primary/secondary care interface might facilitate improved management of people with HFpEF. This paper complements our qualitative paper based on interviews with specialist care providers, patients and carers, and adds further insights into GP views on identifying and managing people with HFpEF.^52^

### Strengths and limitations

This paper is part of a larger programme of work specifically exploring the challenges faced by GPs in managing people with HFpEF in UK primary care. Data were collected from GPs in rural, suburban and urban areas in three different regions in England. Purposive sampling ensured we included GPs with a range of experience, working within different communities including exploring care for people from BAME groups. Data collection was facilitated by a topic guide but allowed participants to talk openly about day-to-day challenges of managing people with HFpEF.

We conducted both face-to-face and telephone interviews. It is suggested that telephone interviews may be a less effective way of generating data than face-to-face interviews, due to a greater difficulty in achieving rapport, and loss of visual cues.^53^ Other researchers argue that telephone interviews facilitate the disclosure of challenging or sensitive questions where an interviewee might feel more relaxed and unrestricted due to the physical absence of the interviewer.^54,55^ The analysis was conducted by a team of researchers by different backgrounds. Regular team meetings allowed for on-going discussion of transcripts and issues from the reflective notes, and points arising from reflective notes which enabled interviewers to explore emerging concepts in later interviews.

GPs were restricted to three regions in England, so results may not be transferrable to the NHS in England. We did, however, obtain a range of views, with GPs describing interactions with several specialist care services. GPs might have felt that the interview was a test or judgement of their knowledge about HFpEF – some mentioned preparing for the interview by reading about HFpEF. However, the three interviewers had a non-clinical background and thus avoided ‘shared conceptual blindness’.^56^

### Implications for clinical practice

It is important to raise awareness and knowledge about HFpEF to enable GPs to consider and make the diagnosis and support the management of patients in primary care. This will allow for a recognition of the complexity of the condition and provision of personalised care where possible. Special attention is needed to support people with health literacy and language difficulties, such as people form BAME groups. Education and training are essential to increase GPs’ knowledge and skills in considering HFpEF as a possible diagnosis. Communication between primary and specialist care teams is key, with clear referral pathways established to support the management of people with HFpEF.

## Conclusion

This study has shown that GPs may have limited knowledge and understanding about HFpEF and lack the confidence to make the diagnosis and manage those with the condition. Greater integration between primary and secondary care could improve management for people with HFpEF. This study provides evidence to support the development of pathways between primary and secondary care when managing people with HFpEF that can be delivered in primary care.

## Supplemental Material

sj-pdf-1-chi-10.1177_1742395320983871 - Supplemental material for Challenges in the management of people with heart failure with preserved ejection fraction (HFpEF) in primary care: A qualitative study of general practitioner perspectives Click here for additional data file. Supplemental material, sj-pdf-1-chi-10.1177_1742395320983871 for Challenges in the management of people with heart failure with preserved ejection fraction (HFpEF) in primary care: A qualitative study of general practitioner perspectives by Muhammad Z Hossain, Carolyn A Chew-Graham, Emma Sowden, Tom Blakeman, Ian Wellwood, Stephanie Tierney and Christi Deaton in Chronic Illness
