# Supplementary material for: Challenges in the management of people with heart failure with preserved ejection fraction (HFpEF) in primary care: A qualitative study of general practitioner perspectives
Source: Chronic Illn. 2021 Jan 5;18(2):410–25. doi: 10.1177/1742395320983871 (PMC9163769; doi:10.1177/1742395320983871)
Supplement: sj-pdf-1-chi-10.1177_1742395320983871 - Supplemental material for Challenges in the management of people with heart failure with preserved ejection fraction (HFpEF) in primary care: A qualitative study of general practitioner perspectives [file sj-pdf-1-chi-10.1177_1742395320983871.pdf]

Appendices

Appendix 1: Interview Schedule

HFpEF Qualitative study

Healthcare Professionals Interview (v3.0 April 2019)

Study ID____________________

Interview was conducted:

Face to face__ Location______________________________

Telephone__

Other comments____

Interviewer ____

Introduction and your role in care

1. To begin with it would be helpful if you could tell me a bit about your practice / service
   Prompts: size of population, type of patients, special interests, context, staffing
2. Can you tell me about your role in the care of patients with HFpEF ?
   Prompts: Care structures, interface between specialist and primary care, compare to role of others, feelings around self-management
3. Talk me through a bit about patients with HFpEF in your service/practice
   Prompts: How diagnosed and by whom, system of referral, management, titration

   Alternative questions:
   Can you tell me a bit about your experiences of caring for patients with this condition?
   How are people referred into your service? What are the challenges around diagnosis of HFpEF? Communication within the HF service/working with HF nurses.
   What sort of self-management strategies/support do people with HFpEF need to follow?
   How do these experiences compare to patients with other types of heart failure i.e. HFrEF
   What role do you think guidelines play in managing these patients? (ESC 2016, NICE 2010)
   *Can you tell me a bit about your thoughts/views about the term/condition HFpEF (*aim to uncover provider's views/knowledge about term & any scepticism*). What words would you use to explain HFpEF to people with this condition? What message(s) do you think patients take from your explanation?*

Interaction between professionals

4. How do you feel about the information you receive from other HCPs involved in the care of patients with HFpEF? How do you communicate HFpEF back to the GP?

Prompts: Echo results, letters, timely

__Communication__

5. How do you talk about this condition (HFpEF) with patients and carers?

Prompts: Use of the term 'heart failure' (terminology), explaining diagnosis of HFpEF, advice given. *Do you use any euphemisms to name heart failure?*

6. What kind of challenges do you think this people with HFpEF face?

__Ideas for managing the condition__

7. What are your experiences of interventions that seem to work in this patient population?

Prompts: Good practice, in an ideal world; how would the system need to change in order to manage people with HFpEF more effectively?

8. Can you tell me about any challenges you have experienced (or anticipate) when providing services for this patient group?

Prompts: Challenges around emergency admission? Challenges relating to referral criteria to specialist service? Challenges around discharge?

Any particular challenges for any specific patient group, such as ethnic minority group? Explore reasons

__Final question__

8. Is there anything else you want to tell me about management or treatment of patients with HFpEF?

Appendix 2: COREQ checklist

COREQ: Consolidated criteria for reporting qualitative research: a 32-item checklist for interviews and focus groups

Section/Topic	Item No	Checklist item	Reported on page No
**Domain 1: Research team and reflexivity**			
Personal Characteristics			
Interviewer/ Facilitator	1	Which author/s conducted the interview or focus group? Interviewer/facilitator	MH, ES, IW [5, 17]
Credentials	2	What were the researcher's credentials? E.g. PhD, MD	MH: PhD, AFHEA, MA, MSS, BSS.<br>CC-G: BSc, MB ChB, MD, FRCGP.<br>ES: BSc, PhD.<br>TB: PhD, MRCGP.<br>IW: BA, MPhil, PhD, PGCAP, MCSP.<br>ST: PhD, MA, BA.

			CD: PhD, RN, FAHA, FESC, FAAN.
Occupation	3	What was their occupation at the time of the study?	MH: Health Services Researcher.<br>CC-G: Professor of General Practice Research<br>ES: Research Associate<br>TB: Clinical Senior Lecturer in Primary Care<br>IW: Research Associate<br>ST: Departmental Lecturer and Senior Researcher<br>CD: Florence Nightingale Foundation Clinical Professor of Nursing
Gender	4	Was the researcher male or female?	Researchers who performed the interviews were both male and female
Experience and training	5	What experience or training did the researcher have? Relationship with participants	All three researchers (MH, ES, IW) who performed the interviews have PhDs and have 15+ years of qualitative research experiences of conducting interviews.
Relationship with participants			
Relationship established	6	Was a relationship established prior to study commencement?	No relationship with the participant was established prior to study commencement.
Participant knowledge of the interviewer	7	What did the participants know about the researcher? e.g. personal goals, reasons for doing the research	Letters of invitation, participant information sheets, informed consent and expression of interest forms were sent to potential participants. Therefore, participants understood it was a type of heart failure study called Optimise-HFpEF study and participants had the opportunity to review the documents prior to signing their informed consent forms
Interviewer characteristics	8	What characteristics were reported about the interviewer/facilitator? e.g. Bias, assumptions, reasons and interests in the research topic	MH & ES acknowledged their roles as non-clinical researchers.<br>IW acknowledges his role as a clinical researcher.
**Domain 2: study design**			

Theoretical framework			
Methodological orientation and Theory	9	What methodological orientation was stated to underpin the study? e.g. grounded theory, discourse analysis, ethnography, phenomenology, content analysis	Framework analysis [data analysis, 5]
Participant selection			
Sampling	10	How were participants selected? e.g. purposive, convenience, consecutive, snowball	Purposive sampling [5, 16]
Method of approach	11	How were participants approached? e.g. face-to-face, telephone, mail, email	Email, telephone, mail
Sample size	12	How many participants were in the study?	35 [page 2, 6]
Non-participation	13	How many people refused to participate or dropped out? Reasons?	Keele: None dropped out, 2 GPs agreed to be interviewed/contacted MH after we decided to close the interviews/data saturated.
Setting of data collection	14	Where was the data collected? e.g. home, clinic, workplace	General Practices and GPs' workplaces (university)
Presence of non-participants	15	Was anyone else present besides the participants and researchers?	No
Description of sample	16	What are the important characteristics of the sample? e.g. demographic data, date	Demographic data: descriptions of other relevant data such as GP type, length of years in the profession, Practice Location [table 1, page 6]
Data collection			
Interview guide	17	Were questions, prompts, guides provided by the authors? Was it pilot tested?	Yes
Repeat interviews	18	Were repeat interviews carried out? If yes, how many?	No
Audio/visual recording	19	Did the research use audio or visual recording to collect the data?	All interviews were digitally recorded with participants consent [2, 5]
Field notes	20	Were field notes made during and/or after the interview or focus group?	Yes [6]
Duration	21	What was the duration of the interviews or focus group?	Variable duration from 17 minutes to up to 51 minutes [6]
Data saturation	22	Was data saturation discussed?	Yes [data collection, 5]
Transcripts returned	23	Were transcripts returned to participants for comment and/or correction?	No
**Domain 3: analysis and findings**			
Data analysis			

Number of data coders	24	How many data coders coded the data?	7 [Contributors, 17]
Description of the coding tree	25	Did authors provide a description of the coding tree?	No
Derivation of themes	26	Were themes identified in advance or derived from the data?	Derived from the data [7-14]
Software	27	What software, if applicable, was used to manage the data?	NVivo 12 [2, 6]
Participant checking	28	Did participants provide feedback on the findings?	N/A
Reporting			
Quotations presented	29	Were participant quotations presented to illustrate the themes / findings? Was each quotation identified? e.g. participant number	Yes, participant number [7-14]
Data and findings consistent	30	Was there consistency between the data presented and the findings?	Yes [7-14]
Clarity of major themes	31	Were major themes clearly presented in the findings?	Yes [7-14]
Clarity of minor themes	32	Is there a description of diverse cases or discussion of minor themes?	Yes [BAME communities]
